# Surgically Induced Contrast Enhancements on Intraoperative and Early Postoperative MRI Following High-Grade Glioma Surgery: A Systematic Review

**DOI:** 10.3390/diagnostics11081344

**Published:** 2021-07-26

**Authors:** Alexander Malcolm Rykkje, Dana Li, Jane Skjøth-Rasmussen, Vibeke Andrée Larsen, Michael Bachmann Nielsen, Adam Espe Hansen, Jonathan Frederik Carlsen

**Affiliations:** 1 Department of Diagnostic Radiology, Copenhagen University Hospital, Rigshospitalet, 2100 Copenhagen, Denmark; dana.li@regionh.dk (D.L.); vibeke.andree.larsen@regionh.dk (V.A.L.); mbn@dadlnet.dk (M.B.N.); adam.espe.hansen@regionh.dk (A.E.H.); jonathan.frederik.carlsen@regionh.dk (J.F.C.); 2 Department of Clinical Medicine, University of Copenhagen, 2200 Copenhagen, Denmark; 3 Department of Neurosurgery, Copenhagen University Hospital, Rigshospitalet, 2100 Copenhagen, Denmark; jane.skjoeth-rasmussen@regionh.dk

**Keywords:** high-grade glioma, intraoperative MRI, early postoperative MRI, postoperative enhancement, time window

## Abstract

For the radiological assessment of resection of high-grade gliomas, a 72-h diagnostic window is recommended to limit surgically induced contrast enhancements. However, such enhancements may occur earlier than 72 h post-surgery. This systematic review aimed to assess the evidence on the timing of the postsurgical MRI. PubMed, Embase, Web of Science and Cochrane were searched following Preferred Reporting Items for Systematic Reviews and Meta-Analyses (PRISMA) guidelines. Only original research articles describing surgically induced contrast enhancements on MRI after resection for high-grade gliomas were included and analysed. The frequency of different contrast enhancement patterns on intraoperative MRI (iMRI) and early postoperative MRI (epMRI) was recorded. The search resulted in 1443 studies after removing duplicates, and a total of 12 studies were chosen for final review. Surgically induced contrast enhancements were reported at all time points after surgery, including on iMRI, but their type and frequency vary. Thin linear contrast enhancements were commonly found to be surgically induced and were less frequently recorded on postoperative days 1 and 2. This suggests that the optimal time to scan may be at or before this time. However, the evidence is limited, and higher-quality studies using larger and consecutively sampled populations are needed.

## 1. Introduction

High-grade gliomas are the most common malignant brain tumours in adults and are generally difficult to treat and have a poor prognosis [1,2]. Survival is correlated with the extent of surgical resection, which is usually evaluated on magnetic resonance imaging (MRI) soon after surgery [3,4]. Such an assessment is often difficult to make as both the surgical procedure and residual tumour tissue can cause contrast enhancements on MRI [5]. Differentiating between surgically induced contrast enhancements and residual tumours can be difficult and requires careful comparison with the preoperative and follow-up scans [6]. Other surgically induced changes such as T1-weighted hyperintensities caused by methaemoglobin and areas of restricted diffusion caused by ischemia can also be difficult to distinguish from residual tumour, but are less discussed in the literature [7,8].

Today, the recommendation is to perform the early postoperative MRI (epMRI) within 72 h after surgery [9,10]. The evidence for this diagnostic window is based on only a handful of studies from the 1990s [11,12,13,14]. Later studies found that such surgically induced enhancements may occur earlier than the 72-h window [15,16]. In a recent study, these changes have been seen to develop as early as 17 h post-surgery [6]. Several studies report benefits from performing MRI as early as in the operating theatre to maximise the gross total resection [17,18,19,20]. However, even on intraoperative MRI (iMRI) there may be surgically induced changes, which can complicate the assessment of the extent of resection [21,22]. The optimal window for scanning high-grade gliomas is therefore still debatable, especially in an era with increased use of iMRI, and indeed in a time where surgery of high-grade gliomas is performed more and more frequently [23].

We performed a systematic review of the literature on the timing of the postsurgical MRI. The evidence of the occurrence of surgically induced contrast enhancement on MRI at different time points during, and after, high-grade glioma surgery was investigated. To the best of our knowledge, no previous systematic review has been done on this topic.

## 2. Materials and Methods

The study research question was created using the PICOS (Patient, Intervention, Comparison, Outcome, Study type) framework [24]. Our research question was as follows: In patients operated for high-grade glioma, which MRI examination time point is most efficient for differentiating surgically induced contrast enhancements from a residual tumour? The time points include intraoperative MRI, and special attention is given to the evidence for the conventional 72-h diagnostic window.

The literature search was performed using the databases of PubMed, Embase, Web of Science and Cochrane, with the last search taking place on 4 May 2021. The search string was optimised for each database.

The PubMed and Cochrane queries with MeSH terms were “magnetic resonance imaging” [MeSH] AND “perioperative period” [MeSH] AND “time factors” [MeSH] AND “glioblastoma” [MeSH]. The Embase query with emtree terms was: “nuclear magnetic resonance imaging” [Emtree Terms] AND (“postoperative period” [Emtree Terms] OR “intraoperative period” [Emtree Terms]) AND “contrast enhancement” [Emtree Terms] AND “glioblastoma” [Emtree Terms]. Additionally, the following keywords were used in all four databases: (“MRI” OR “magnetic resonance” OR “magnetic resonance imaging”) AND (“postoperative” OR “intraoperative” OR “peroperative”) AND (“time” OR “timing” OR “enhancement” OR “enhancements” OR “changes” OR “pattern” OR “patterns”) AND (“glioblastoma” OR “high-grade glioma” OR “malignant glioma” OR “GBM” OR HGG”).

**Figure 1:**
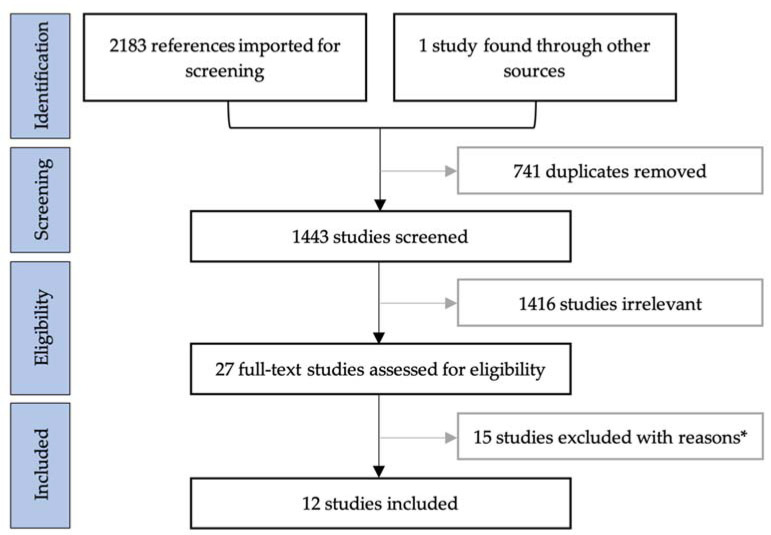
Flow chart of the literature search following Preferred Reporting Items for Systematic Reviews and Meta-Analyses (PRISMA) guidelines. * Reasons for exclusions after full-text assessment provided in Appendix A.

Following PRISMA guidelines (Preferred Reporting Items for Systematic Reviews and Meta-Analyses) [25], duplicates were removed and studies were screened by title and abstract using the web-based programme Covidence. The study selection process is summarised in Figure 1. Peer-reviewed articles of original research in English were included if they had descriptions of surgically induced contrast enhancements on iMRI and/or epMRI for patients with high-grade glioma. No restrictions were put on the publication date or length of follow-up. Reviews, case reports and articles with overlapping data were excluded. Full-text articles were assessed using the same criteria. Reasons for excluding articles after full-text assessments are provided in Appendix A.

For the data collection, information was gathered in a spreadsheet and organised into categories including, but not limited to, study design, study population, MRI parameters, timing as well as methods and results for assessing surgically induced contrast enhancement. Articles were independently assessed for risk of bias by two authors (A.M.R. and J.F.C.). To assess bias, modified items from the QUADAS tool (Quality Assessment of Diagnostic Accuracy Studies) were used in an informal manner. A meta-analysis was not suitable since we did not find sufficient data for quantitative analysis.

## 3. Results

### 3.1. Study Characteristics

Twelve studies were included for qualitative synthesis. For details on the methodology, please see Table 1. Six studies were retrospective studies [6,15,16,21,26,27], and six were prospective studies [11,12,28,29,30,31]. No randomised controlled studies met the inclusion criteria. The included studies were published between 1993 and 2020. Four studies involved intraoperative MRI [21,28,29,30], while the remaining eight studies had a postoperative focus [6,11,12,15,16,26,27,31]. Five studies recruited patients with high-grade gliomas only. Of these five, only three solely included glioblastoma patients [6,12,26]. Six studies also included low-grade gliomas or metastases as well as high-grade gliomas [15,21,27,28,29,30]. One of these studies specified their results for high-grade gliomas [27]. The field strengths varied greatly in the included studies: from low field iMRI scanners using 0.2 Tesla (T) in early studies to high-field 3 T scanners in the more recent studies.

**Figure 2:**
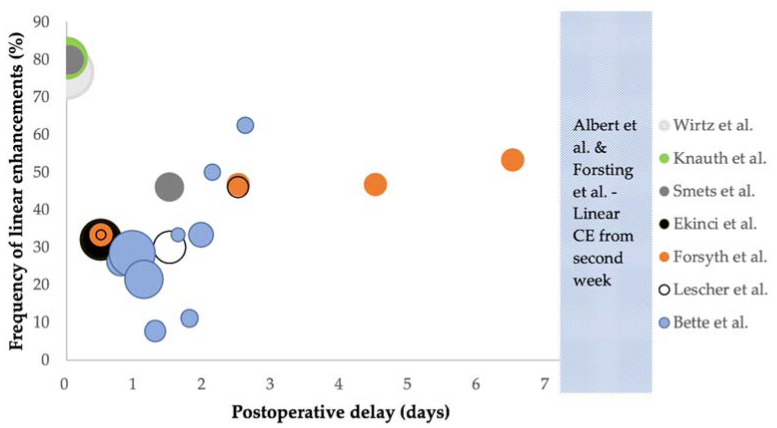
Bubble chart showing the total frequency of linear enhancements (*y*-axis) at different time intervals during or after surgery (*x*-axis), with the median of each reported time interval registered on the *x*-axis. The size of the bubble represents the total number of patients who had MRI performed, up to 86 patients for Wirtz et al. [29]. Each study is presented with a different colour as shown in the legend. Plots for Ekinci et al. [15], Lescher et al. [16] and Forsyth et al. [31] were calculated by the authors of this review using data from each paper. All grades of linear enhancement are shown for Forsyth et al. Abbreviations: CE, contrast enhancement.

Contrast enhancements were in most cases classified by their enhancement pattern on MRI (10/12 studies) [6,11,12,15,16,26,27,29,30,31]. The enhancement pattern most frequently associated with surgically induced changes was linear enhancement [6,11,12,15,16,26,29,30,31]. In studies where linear enhancement is mentioned, the frequency was recorded in all but two studies [11,12]. These are presented in Table 2 and Figure 2. For a summary of other enhancement patterns, please see Appendix B. In two studies, there were no descriptions of morphology [21,28]. Instead, contrast enhancements that were not present on the preoperative scan were described as new procedure-related enhancements.

Contrast enhancements were categorised as surgically induced, or as residual tumour, by comparing with either preoperative imaging (4/12 studies) [21,28,29,30] or follow-up imaging (8/12 studies) [6,11,12,15,16,26,27,31]. In general, the contrast enhancements that were predominantly correlated to a residual or recurring tumour were nodular, thick linear or frayed in appearance. Conversely, for all studies recording linear enhancement, this was predominantly related to surgically induced contrast enhancement.

### 3.2. Results of Individual Studies

#### 3.2.1. Studies on Intraoperative MRI

Miskin et al. and Masuda et al. reported data on iMRI compared with epMRI and recorded surgically induced contrast enhancements as new enhancements without specifying their morphology [21,28]. Miskin et al. reported new enhancements in 16% (10/64) on iMRI. Of the patients who had an epMRI (7/10), all new enhancements recorded on iMRI had either decreased (5/7) or resolved (2/7) on epMRI [21]. Masuda et al. reported new enhancements as present in 36.4% (8/22) on iMRI and in 54.5% (12/22) on epMRI [28]. Wirtz et al. and Knauth et al. recorded the intraoperative frequency of surgically induced contrast enhancements on iMRI as compared with preoperative MRI [29,30]. Wirtz et al. found linear enhancements occurring in 76.7% (66/86) [29]. Knauth et al. included descriptive comparisons with epMRI on postoperative days 1–3 but provided no data. On iMRI, the frequency of linear enhancement along the resection margins was 80.4% (41/51), but this decreased or resolved on epMRI [30].

#### 3.2.2. Studies on Early Postoperative MRI within 72 h

Four studies reported data on early postoperative MRI obtained within 72 h after surgery [6,15,16,26]. Smets et al. recorded linear enhancement in 80% within 2 h, and in 46% at 24–48 h after surgery among 24 patients, but did not reach statistical significance for correlation with recurrence [26]. Ekinci et al. identified thin linear enhancements on epMRI within 24 h after surgery for 16 patients (32%), and tumour recurrence did not develop on follow-up imaging in 14 of those patients [15]. Bette et al. had linear enhancement appear in 24.1% (39/162) before 45 h, and after 45 h in 45.5% (20/44). The study found that reactive enhancement appeared in 17.9% (29/162) within 45 h, and in 34.1% (15/44) after 45 h when comparing with follow-up imaging. Linear enhancement was more often reactive (66.1%, 39/59), and nodular enhancement mostly represented residual tumour (93.2%, 68/73) [6]. In Lescher et al., one of three patients presented with surgically induced contrast enhancement at <24 h, 30% (9/30) of patients between 24 and 48 h, and 46% (6/13) of patients beyond 48 h. Surgically induced contrast enhancement was seen within 72 h in 28.3% (13/46), and as early as 22:57 h after surgery [16].

#### 3.2.3. Studies on Postoperative MRI beyond 72 h

Four studies reported data that included postoperative MRI performed beyond 72 h [11,12,27,31]. While Sui et al. provided no data for the different time intervals, the overall incidence of surgically induced contrast enhancement was 52.9% (37/70) for patients with high-grade gliomas, with the most significant enhancement seen 6–30 days after surgery [27]. Forsyth et al. recorded linear enhancement graded 2–3 in intensity occurred in 0% (0/15) on day 1, 20% (3/15) on day 3, 40% (6/15) on day 5, 40% (6/15) on day 7, 53.3% (8/15) on day 14, and 27% (4/15) on day 21, with the highest frequency recorded on days 5 to 14 [31]. Albert et al. [11] and Forsting et al. [12] did not record any data for surgically induced contrast enhancements. Instead, observations were done descriptively. Albert et al. reported linear enhancement occasionally appearing on day 4 but not before that [11]. Beginning in the second postoperative week, both studies recorded widespread linear enhancement, which evolved rapidly after that time and persisted for up to 2 months. 

### 3.3. Bias Assessment

There is limited information on blinding in the included studies. Three studies made use of two or more independent interpreters, where agreement was reached by discussion or majority [11,21,28]. In the remaining studies, there was either one interpreter [26,31], no blinding between interpreters [6,15,16,29] or no information on interpreters [12,27,30]. In two studies, interpreters were blinded to the results of follow-up imaging [16,26], and in one study, interpreters were blinded to the time between surgery and imaging [6]. A lack of blinding could increase the risk of bias, in particular for observational studies with subjective outcomes, such as when interpreting images. This variability could lead to observer bias. Finally, in eight of the 12 included studies it is not clear whether patients were consecutively sampled, in which case selection bias cannot be ruled out [11,12,15,16,26,27,30,31].

## 4. Discussion

The studies presented in this review show that surgically induced contrast enhancement can appear at all time points after surgery, as well as on intraoperative MRI (iMRI), but their type and frequency vary. Thin linear contrast enhancements are often surgically induced and seem to occur less frequently on postoperative days 1 and 2, suggesting that the optimal time to scan could be at or before this time. 

The evidence provided for the 72-h window is based on studies from the 1990s [11,13,14]. Albert et al. were the first to mention a “diagnostic window” which exists 3 days after surgery, after which surgically induced contrast enhancement would develop and could remain present for weeks [11]. Some of these early studies recruited patients with non-neoplastic conditions that do not enhance on MRI [13,14]. Enhancement appeared predominantly as thin linear on days 0 to 5, changing to predominantly thick linear or nodular enhancement from day 6, making it increasingly difficult to differentiate from residual tumour. Based on these early studies, current guidelines recommend performing the postoperative MRI between 24 and 48 h after surgery, and no later than 72 h after surgery [9]. Similarly, a recent study analysed the postoperative window in association with survival using image processing and radiomics. Postoperative enhancement thickness correlated with survival on scans performed between 24 and 72 h after surgery [32].

Since the frequency and severity of surgically induced enhancements increase with time, pre-radiotherapy MRI (prMRI) performed 2 to 8 weeks after surgery is generally not used to assess the extent of resection. However, recent studies have suggested that prMRI could serve as a more accurate baseline for later follow-up imaging, in particular for predicting overall survival [33,34].

The early postoperative MRI mainly serves two purposes. One is to determine the extent of resection following surgery, and the other is as a baseline for future assessments of recurrence. For both purposes, recognising when contrast enhancements are caused by residual tumours or induced by surgery can be difficult and is the main diagnostic challenge addressed in this review. However, surgically induced contrast enhancements do not appear as easily detected predefined types of enhancements. Instead, careful comparisons with preoperative and follow-up imaging are needed for clear differentiations.

In most of the included studies, surgically induced contrast enhancements were described as linear. In two of the included studies, linear enhancements were found to be surgically induced in between 61.5% and 87.5% of the cases [6,15]. The overall frequency of linear enhancement varies greatly between the studies, as Table 2 and Figure 2 demonstrate. Linear enhancements seems to be more frequent during and immediately after surgery (<2 h) with a frequency of up to 80.4% [26,29,30]. Bette et al. included most patients and found linear enhancement to be less frequent before 45 h after surgery (24.1%) as opposed to beyond 45 h (45.5%) [6].

Few studies are available on the use of iMRI for limiting surgically induced contrast enhancement. The studies in this review present diverging results. For example, Miskin et al. and Masuda et al. [21,28] reported a much lower frequency of surgically induced contrast enhancements than Knauth et al. and Wirtz et al. did [29,30], but these contrast enhancements generally seemed to decrease or resolve on epMRI performed beyond 24 h after surgery. While iMRI-guided resection has been shown to improve the extent of resection [20,35], the results presented here suggest that further studies should investigate whether iMRI can be considered a replacement for epMRI.

### 4.1. Limitations of Included Studies

A major limitation of the included studies lies in their heterogeneity. While all studies included in this review dealt with the presence of surgically induced contrast enhancements in some form, they varied in several ways. Significant differences were seen in the number of patients, field strengths and methods for assessing morphology. It is not always clear what changes are reported as surgically induced, and findings on MRI could potentially be interpreted differently by different authors as either surgically induced contrast enhancements, residual tumour or something else. While some of these differences could be due to the widely differing publication years of the included studies, this heterogeneity makes any comparisons between the studies difficult.

### 4.2. Limitations of This Review

The study populations all included, but were not limited to, high-grade gliomas. Most studies did not provide separate results for high-grade gliomas, and due to the limited number of available articles, results consisting of both high- and low-grade gliomas were included in this review. Since there are substantial differences in morphology and patient survival between different grades of gliomas, this can be problematic for the interpretation of the results. However, most patients had high-grade gliomas (87%), while a smaller proportion (13%) had low-grade gliomas.

Assessing risk of bias using modified items from the QUADAS tool in an informal manner is a further limitation of this review. However, for the nonrandomised and noninterventional observational studies in this review, there is no universally accepted standard for assessing the risk of bias [36]. Consequently, there is a significant risk of bias in the included studies.

## 5. Conclusions

In conclusion, surgically induced contrast enhancements can occur at all time points after surgery, and as early as on intraoperative MRI (iMRI), but their type and frequency vary. Thin linear enhancements seem to occur less frequently on postoperative days 1 and 2, suggesting that the optimal time to scan may be at or before this time. However, due to the heterogeneity of the studies, results presented in this review are not conclusive. For this, higher-quality studies using larger and consecutively sampled populations are needed. 

## Figures and Tables

**Table 1:** Methodology of the included studies.

	Authors	Year	Design	Cases	Pathology (%)	Tesla	Sequences	epMRI Timing	CE Assessments	CE Comparison
iMRI	Miskin et al. [21]	2019	R	64	HGG (69%)	3 T iMRI 1.5/3 T epMRI	iMRI: T1 epMRI: NS	<72 h	New enhancements	iMRI and epMRI with preMRI epMRI with iMRI and follow-up
	Masuda et al. [28]	2018	*p*	22	HGG (95%)	1.5 T iMRI 1.5 T epMRI	T1, T2, DWI, MPRAGE	<24 h	New enhancements	PreMRI
	Wirtz et al. [29]	2000	*p*	88	HGG (70%)	0.2 T	T1, T2, FLAIR	*n*/A	Surgically induced as linear, intraparenchymal	PreMRI
	Knauth et al. [30]	1999	*p*	48	HGG (71%)	0.2 T	T1	Day 1–3	Surgically induced as linear, intraparenchymal	PreMRI and epMRI
<72 h	Smets et al. [26]	2013	R	24	GBM (100%)	3 T	T1, T2, DWI	<2 h and24–48 h	Linear, micronodular, frayed	Follow-up MRI
	Ekinci et al. [15]	2003	R	50	HGG (78%)	1.5 T	T1, T2	<24 h	Thin linear, thick linear, thick linear-nodular	Follow-up MRI
	Bette et al. [6]	2016	R	173	GBM (100%)	3 T	T1, FLAIR, MPRAGE	<17 to >72 h in 4-h intervals	Linear, nodular	Follow-up MRI
	Lescher et al. [16]	2014	R	46	HGG (100%)	3 T	T1, T2, FLAIR, other	<24 to >48 h in 3 groups	Surgically induced if thin linear Tumoral if bulky/nodular	Follow-up MRI
>72 h	Sui et al. [27]	2020	R	106	HGG (66%)	3 T	T1, T2, FLAIR	<24 h to 30 days	Enhancement ring	Follow-up MRI
	Forsyth et al. [31]	1997	*p*	17	HGG (100%)	1.5 T (4 on 0.5 T)	T1, T2	Day 1, 3, 5, 7, 14 and 21	Surgically induced as linear. Subdivided by intensity.	Follow-up MRI
	Albert et al. [11]	1994	*p*	60	HGG (100%)	1 T	T1	Day 1–5, week 2, week 4–6, then bimonthly	Enhancement patterns evaluated over time.	Follow-up MRI
	Forsting et al. [12]	1993	*p*	68	GBM (100%)	1 T	T1	Day 1–5, week 2, week 4–6, then bimonthly	Enhancement patterns evaluated over time.	Follow-up MRI

Abbreviations: iMRI, intraoperative magnetic resonance imaging; epMRI, early postoperative magnetic resonance imaging; preMRI, preoperative magnetic resonance imaging; T, tesla; CE, contrast enhancement; NS, not specified; R, retrospective; *p*, prospective; HGG, high-grade glioma; LGG, low-grade glioma; GBM, glioblastoma.

**Table 2:** Frequency of linear enhancements.

	Author	Timing	Frequency (%)		Surgically Induced (% of Linear CE)
iMRI	Wirtz et al. [29]	iMRI	76.7% (66/86)		NS
	Knauth et al. [30]	iMRI	80.4% (41/51)		NS
<72 h	Smets et al. [26]	<2 h 24–48 h	80% 46%		NS
	Ekinci et al. [15]	<24 h	32% (16/50)		87.5% (14/16) *
	Bette et al. [6]	<45 h >45 h	24.1% (39/162) 45.5% (20/44)		61.5% (24/39) 75% (15/20)
	Lescher et al. [16]	<72 h	28.3% (13/46)		NS
>72 h	Forsyth et al. [31]	Day 1 Day 3 Day 5 Day 7 Day 14 Day 21	**All grades** 33.3% (5/15) * 46.7% (7/15) * 46.7% (7/15) * 53.3% (8/15) * 53.3% (8/15) * 40% (6/15) *	**Grade 2–3** 0% (0/15) * 20% (3/15) * 40% (6/15) * 40% (6/15) * 53.3% (8/15) * 27% (4/15) *	NS
	Albert et al. [11]	Day 1–5, week 2, week 4–6, then bimonthly	Did not occur before day 4, developed in week 2 and had resolved after 2 months in most patients		NS
	Forsting et al. [12]	Day 1–5, week 2, week 4–6, then bimonthly	Did not occur before day 4, developed in week 2 and had resolved after 2 months in most patients		NS

* Numbers calculated by authors of this review using data from the included studies. Abbreviations: iMRI, intraoperative magnetic resonance imaging; CE, contrast enhancement; NS, not specified.

## Appendix A

**Table A1:** Reasons for exclusions after full-text assessment.

Author	Reason for Exclusion
Aprile et al., (2008) [37]	Only perfusion MRI
Belhawi et al., (2010) [8]	Only T2-weighted/FLAIR MRI
Boyett et al., (2019) [38]	Conference abstract
Brochado et al., (2012) [39]	Conference abstract
Colen et al., (2012) [40]	Abstract only
Fei et al., (2020) [41]	Only diffusion/perfusion MRI
Finck et al., (2020) [42]	Only black-blood MRI sequence
Florez et al., (2020) [43]	Conference abstract
Garcia-Ruiz et al., (2021) [32]	No data for patterns of contrast enhancements
Heßelmann et al., (2017) [44]	No data for patterns of contrast enhancements
Lescher et al., (2016) [45]	Only FLAIR MRI
Lescher et al., (2014) [46]	Conference abstract
Majos et al., (2016) [5]	No data for patterns of contrast enhancements
Martin et al., (2000) [47]	No data for patterns of contrast enhancements
Özduman et al., (2014) [48]	Only dynamic contrast enhanced (DCE)-MRI

## Appendix B

**Table A2:** Other patterns of contrast enhancements.

Authors	Enhancement Patterns					
	Meningeal	Increased Choroid Plexus	Intraparenchymal	Enhancement Ring	Nodular	Frayed
Wirtz et al. [29]	X	X	X			
Knauth et al. [30]	X	X	X			
Smets et al. [26]					X	X
Ekinci et al. [15]					X	
Bette et al. [6]	X				X	
Lescher et al. [16]					X	
Sui et al. [27]	X			X		
Forsyth et al. [31]	X					

## References

[1] Gandhi S., Tayebi Meybodi A., Belykh E., Cavallo C., Zhao X., Syed M.P., Borba Moreira L., Lawton M.T., Nakaji P., Preul M.C. (2019). Survival Outcomes Among Patients with High-Grade Glioma Treated with 5-Aminolevulinic Acid-Guided Surgery: A Systematic Review and Meta-Analysis. Front. Oncol..

[2] Thakkar J.P., Dolecek T.A., Horbinski C., Ostrom Q.T., Lightner D.D., Barnholtz-Sloan J.S., Villano J.L. (2014). Epidemiologic and Molecular Prognostic Review of Glioblastoma. Cancer Epidemiol. Biomark. Prev..

[3] Stummer W., Reulen H.-J., Meinel T., Pichlmeier U., Schumacher W., Tonn J.-C., Rohde V., Oppel F., Turowski B., Woiciechowsky C. (2008). Extent of Resection and Survival in Glioblastoma Multiforme: Identification of and Adjustment for Bias. Neurosurgery.

[4] Grabowski M.M., Recinos P.F., Nowacki A.S., Schroeder J.L., Angelov L., Barnett G.H., Vogelbaum M.A. (2014). Residual Tumor Volume versus Extent of Resection: Predictors of Survival after Surgery for Glioblastoma. J. Neurosurg..

[5] Majos C., Cos M., Castaner S., Gil M., Plans G., Lucas A., Bruna J., Aguilera C. (2016). Early Post-Operative Magnetic Resonance Imaging in Glioblastoma: Correlation among Radiological Findings and Overall Survival in 60 Patients. Eur. Radiol..

[6] Bette S., Gempt J., Huber T., Boeckh-Behrens T., Ringel F., Meyer B., Zimmer C., Kirschke J.S. (2016). Patterns and Time Dependence of Unspecific Enhancement in Postoperative Magnetic Resonance Imaging After Glioblastoma Resection. World Neurosurg..

[7] Ulmer S., Braga T.A., Barker F.G., Lev M.H., Gonzalez R.G., Henson J.W. (2006). Clinical and Radiographic Features of Peritumoral Infarction Following Resection of Glioblastoma. Neurology.

[8] Belhawi S.M.K., Hoefnagels F.W.A., Baaijen J.C., Aliaga E.S., Reijneveld J.C., Heimans J.J., Barkhof F., Vandertop W.P., Hamer P.C.D.W. (2011). Early Postoperative MRI Overestimates Residual Tumour after Resection of Gliomas with No or Minimal Enhancement. Eur. Radiol..

[9] Vogelbaum M.A., Jost S., Aghi M.K., Heimberger A.B., Sampson J.H., Wen P.Y., Macdonald D.R., Van den Bent M.J., Chang S.M. (2012). Application of Novel Response/Progression Measures for Surgically Delivered Therapies for Gliomas: Response Assessment in Neuro-Oncology (RANO) Working Group. Neurosurgery.

[10] Thust S.C., Heiland S., Falini A., Jäger H.R., Waldman A.D., Sundgren P.C., Godi C., Katsaros V.K., Ramos A., Bargallo N. (2018). Glioma Imaging in Europe: A Survey of 220 Centres and Recommendations for Best Clinical Practice. Eur. Radiol..

[11] Albert F.K., Forsting M., Sartor K., Adams H.P., Kunze S. (1994). Early Postoperative Magnetic Resonance Imaging after Resection of Malignant Glioma: Objective Evaluation of Residual Tumor and Its Influence on Regrowth and Prognosis. Neurosurgery.

[12] Forsting M., Albert F.K., Kunze S., Adams H.P., Zenner D., Sartor K. (1993). Extirpation of Glioblastomas: MR and CT Follow-up of Residual Tumor and Regrowth Patterns. AJNR Am. J. Neuroradiol..

[13] Sato N., Bronen R.A., Sze G., Kawamura Y., Coughlin W., Putman C.M., Spencer D.D. (1997). Postoperative Changes in the Brain: MR Imaging Findings in Patients without Neoplasms. Radiology.

[14] Henegar M.M., Moran C.J., Silbergeld D.L. (1996). Early Postoperative Magnetic Resonance Imaging Following Nonneoplastic Cortical Resection. J. Neurosurg..

[15] Ekinci G., Akpinar I.N., Baltacioğlu F., Erzen C., Kiliç T., Elmaci I., Pamir N. (2003). Early-Postoperative Magnetic Resonance Imaging in Glial Tumors: Prediction of Tumor Regrowth and Recurrence. Eur. J. Radiol..

[16] Lescher S., Schniewindt S., Jurcoane A., Senft C., Hattingen E. (2014). Time Window for Postoperative Reactive Enhancement after Resection of Brain Tumors: Less than 72 h. Neurosurg. Focus.

[17] Knauth M., Wirtz C.R., Tronnier V.M., Aras N., Kunze S., Sartor K. (1999). Intraoperative MR Imaging Increases the Extent of Tumor Resection in Patients with High-Grade Gliomas. AJNR Am. J. Neuroradiol..

[18] Kuhnt D., Becker A., Ganslandt O., Bauer M., Buchfelder M., Nimsky C. (2011). Correlation of the Extent of Tumor Volume Resection and Patient Survival in Surgery of Glioblastoma Multiforme with High-Field Intraoperative MRI Guidance. Neuro-Oncology.

[19] Schneider J.P., Trantakis C., Rubach M., Schulz T., Dietrich J., Winkler D., Renner C., Schober R., Geiger K., Brosteanu O. (2005). Intraoperative MRI to Guide the Resection of Primary Supratentorial Glioblastoma Multiforme—A Quantitative Radiological Analysis. Neuroradiology.

[20] Senft C., Bink A., Franz K., Vatter H., Gasser T., Seifert V. (2011). Intraoperative MRI Guidance and Extent of Resection in Glioma Surgery: A Randomised, Controlled Trial. Lancet Oncol..

[21] Miskin N., Unadkat P., Carlton M.E., Golby A.J., Young G.S., Huang R.Y. (2019). Frequency and Evolution of New Postoperative Enhancement on 3 Tesla Intraoperative and Early Postoperative Magnetic Resonance Imaging. Neurosurgery.

[22] Zaidi H.A., Chowdhry S.A., Wilson D.A., Spetzler R.F. (2014). The Dilemma of Early Postoperative Magnetic Resonance Imaging: When Efficiency Compromises Accuracy: Case Report. Neurosurgery.

[23] Lara-Velazquez M., Al-Kharboosh R., Jeanneret S., Vazquez-Ramos C., Mahato D., Tavanaiepour D., Rahmathulla G., Quinones-Hinojosa A. (2017). Advances in Brain Tumor Surgery for Glioblastoma in Adults. Brain Sci..

[24] Eriksen M.B., Frandsen T.F. (2018). The Impact of Patient, Intervention, Comparison, Outcome (PICO) as a Search Strategy Tool on Literature Search Quality: A Systematic Review. J. Med. Libr. Assoc. JMLA.

[25] Moher D., Liberati A., Tetzlaff J., Altman D.G., PRISMA Group (2009). Preferred Reporting Items for Systematic Reviews and Meta-Analyses: The PRISMA Statement. Ann. Intern. Med..

[26] Smets T., Lawson T.M., Grandin C., Jankovski A., Raftopoulos C. (2013). Immediate Post-Operative MRI Suggestive of the Site and Timing of Glioblastoma Recurrence after Gross Total Resection: A Retrospective Longitudinal Preliminary Study. Eur. Radiol..

[27] Sui Z., Zhang X., Li H., Xu D., Li G. (2020). Magnetic Resonance Imaging Evaluation of Brain Glioma before Postoperative Radiotherapy. Clin. Transl. Oncol..

[28] Masuda Y., Akutsu H., Ishikawa E., Matsuda M., Masumoto T., Hiyama T., Yamamoto T., Kohzuki H., Takano S., Matsumura A. (2019). Evaluation of the Extent of Resection and Detection of Ischemic Lesions with Intraoperative MRI in Glioma Surgery: Is Intraoperative MRI Superior to Early Postoperative MRI?. J. Neurosurg..

[29] Wirtz C.R., Knauth M., Staubert A., Bonsanto M.M., Sartor K., Kunze S., Tronnier V.M. (2000). Clinical Evaluation and Follow-up Results for Intraoperative Magnetic Resonance Imaging in Neurosurgery. Neurosurgery.

[30] Knauth M., Aras N., Wirtz C.R., Dörfler A., Engelhorn T., Sartor K. (1999). Surgically Induced Intracranial Contrast Enhancement: Potential Source of Diagnostic Error in Intraoperative MR Imaging. AJNR Am. J. Neuroradiol..

[31] Forsyth P.A., Petrov E., Mahallati H., Cairncross J.G., Brasher P., MacRae M.E., Hagen N.A., Barnes P., Sevick R.J. (1997). Prospective Study of Postoperative Magnetic Resonance Imaging in Patients with Malignant Gliomas. J. Clin. Oncol..

[32] Garcia-Ruiz A., Naval-Baudin P., Ligero M., Pons-Escoda A., Bruna J., Plans G., Calvo N., Cos M., Majós C., Perez-Lopez R. (2021). Precise Enhancement Quantification in Post-Operative MRI as an Indicator of Residual Tumor Impact Is Associated with Survival in Patients with Glioblastoma. Sci. Rep..

[33] De Barros A., Attal J., Roques M., Nicolau J., Sol J.-C., Charni S., Cohen-Jonathan-Moyal E., Roux F.-E. (2020). Glioblastoma Survival Is Better Analyzed on Preradiotherapy MRI than on Postoperative MRI Residual Volumes: A Retrospective Observational Study. Clin. Neurol. Neurosurg..

[34] Booth T.C., Luis A., Brazil L., Thompson G., Daniel R.A., Shuaib H., Ashkan K., Pandey A. (2020). Glioblastoma Post-Operative Imaging in Neuro-Oncology: Current UK Practice (GIN CUP Study). Eur. Radiol..

[35] Kubben P.L., ter Meulen K.J., Schijns O.E., ter Laak-Poort M.P., van Overbeeke J.J., van Santbrink H. (2011). Intraoperative MRI-Guided Resection of Glioblastoma Multiforme: A Systematic Review. Lancet Oncol..

[36] Bero L., Chartres N., Diong J., Fabbri A., Ghersi D., Lam J., Lau A., McDonald S., Mintzes B., Sutton P. (2018). The Risk of Bias in Observational Studies of Exposures (ROBINS-E) Tool: Concerns Arising from Application to Observational Studies of Exposures. Syst. Rev..

[37] Aprile I., Armadori M., Conti G., Ottaviano I., Ranaldi A., Ottaviano P. (2008). MR Perfusion Imaging of Intracranial Tumors: A Retrospective Study of 218 Patients. Neuroradiol. J..

[38] Boyett D., Englander Z., Zanazzi Z., Marie T., McKhann G., Sisti M., Grinband J., Canoll P., Bruce J. (2019). MR Imaging Is Not Reliable for Tumor Presence in Post-Treatment Recurrent High-Grade Glioma. J. Neurosurg..

[39] Brochado A.T.V.H.S.R., Reis C., Linhares P., Rocha A., Vaz R. (2012). Early Postoperative Magnetic Resonance Imaging in Glioblastomas. Neuroradiology.

[40] Colen R., Kovacs A., Zinn P., Jolesz F. (2012). MRI to Predict Surgical and Radiation Dosimetry Borders: A Methodology Feasibility Study. Neuro-Oncology.

[41] Fei Q., Qian L.-X., Zhang Y.-J., Guo W.-J., Bian X.-H., Yin L., Yan P.-W., Wang T.-T., Qian P.-D., Guo Z. (2020). The Utility of Diffusion and Perfusion Magnetic Resonance Imaging in Target Delineation of High-Grade Gliomas. BioMed Res. Int..

[42] Finck T., Gempt J., Krieg S.M., Meyer B., Zimmer C., Wiestler B., Kirschke J.S., Sollmann N. (2020). Assessment of the Extent of Resection in Surgery of High-Grade Glioma—Evaluation of Black Blood Sequences for Intraoperative Magnetic Resonance Imaging at 3 Tesla. Cancers.

[43] Florez E., Hamidi R., Howard C. (2020). Response Assessment in Recurrent Glioblastoma Based on Contrast-Enhanced T1-Weighted Subtraction Color Maps and Rano Criteria. J. Investig. Med..

[44] Hesselmann V., Mager A.-K., Goetz C., Detsch O., Theisgen H.-K., Friese M., Schwindt W., Gottschalk J., Kremer P. (2017). Accuracy of High-Field Intraoperative MRI in the Detectability of Residual Tumor in Glioma Grade IV Resections. Rofo.

[45] Lescher S., Jurcoane A., Schniewindt S., Senft C., Hattingen E. (2016). Misleading FLAIR Imaging Pattern after Glioma Surgery with Intraoperative MRI. Neurosurg. Rev..

[46] Lescher S., Schniewindt S., Jurcoane A., Senft S., Hattingen E. (2014). SAH-like Pattern in Flair Imaging after Intraoperative MRI Guidance in Patients with Malignant Gliomas Surgery. Clin. Neuroradiol..

[47] Martin A.J., Hall W.A., Liu H., Pozza C.H., Michel E., Casey S.O., Maxwell R.E., Truwit C.L. (2000). Brain Tumor Resection: Intraoperative Monitoring with High-Field-Strength MR Imaging-Initial Results. Radiology.

[48] Ozduman K., Yildiz E., Dincer A., Sav A., Pamir M.N. (2014). Using Intraoperative Dynamic Contrast-Enhanced T1-Weighted MRI to Identify Residual Tumor in Glioblastoma Surgery. J. Neurosurg..

